# Constant Torque Rolling Resistance Makes Shear Friction Depend on the Time Step Rather than on Particle Stiffness in Ring Shear Simulations of Monodisperse Glass Spheres

**DOI:** 10.3390/ma19214696

**Published:** 2026-11-04

**Authors:** Yi Liu, Yangshuai Zheng

**Affiliations:** College of Intelligent Construction and Manufacturing, Sichuan Technology and Business University, Chengdu 611745, China

**Keywords:** discrete element method, particle stiffness, time step, rolling resistance, ring shear, glass beads, shear friction, contact network

## Abstract

Discrete element models of glass beads are routinely run with particles two to three orders of magnitude softer than real glass, and softened models usually show lower shear strength, attributed to the added contact compliance. Softening, however, also lengthens the time step allowed by the Rayleigh criterion, so compliance and time step change together. We sheared monodisperse glass spheres with a constant torque rolling resistance in a ring shear cell at 10 kPa, varied the ratio of shear modulus to normal stress *G*/σ_n_ from 10^3^ to 3 × 10^5^, and separated the two effects by restarting runs at 10^3^, 10^4^ and 10^5^ with time steps up to eight times smaller. With Rayleigh-based steps, the friction of the softest grains was 21% below that of the stiffest; at reduced steps, it differed by no more than about 2% across the range, and without rolling resistance it depended on neither quantity. The friction deficit grows with the angular impulse that the rolling torque imparts in one step, which also produces spurious spin fluctuations. Coordination number, rattler fraction and contact persistence, by contrast, change with the stiffness itself. When constant torque rolling resistance is used, time step convergence must therefore be checked separately from stiffness.

## 1. Introduction

The discrete element method (DEM) follows every grain of a granular material and has become a standard tool for linking particle properties to bulk behavior [1]. Glass beads are its favorite test material: they are nearly spherical, their contact properties are well documented, and they are easy to reproduce in the laboratory. Their stiffness, however, is a practical obstacle. Contacts are usually Hertzian [2,3,4], and an explicit integrator must resolve each collision, so the stable time step scales with the grain size times the square root of density over shear modulus [5,6]. For glass with a shear modulus of about 26 GPa, millimeter grains need time steps below 0.1 µs, and a few seconds of shear require tens of millions of steps. The common remedy is to soften the particles by two to three orders of magnitude, which cuts the cost by a factor of ten to thirty [7,8,9]. Softened particles are then often calibrated against bulk tests or used to compare one set of contact parameters with another [10,11,12].

How far one may soften has been studied from two directions. The distance from the rigid limit is commonly measured by a stiffness number κ, the ratio of the contact modulus to the confining pressure raised to the power 2/3 for Hertzian contacts [13]. Two-dimensional simulations of plane shear found that grain elasticity has little influence on dense flows [14]. In three-dimensional simple shear, however, softness adds a second dimensionless group that breaks the inertial number scaling [15], and the Bagnold scaling of inertial flows holds only above a threshold stiffness that is higher for Hertzian than for linear contacts [16]. In slow shear, softer grains form denser packings and carry a lower effective friction that follows the anisotropy of the contact fabric [17], and quasi-static shear of a soft particle system has also been examined directly [18]. In two-dimensional simulations across the transition from soft to rigid particles, the friction law depends only marginally on stiffness, whereas contact network properties such as the coordination number are affected strongly [19]. Engineering studies, in turn, ask how far the shear modulus can be lowered before the bulk behavior changes [7,8,9], and note that attractive forces must be rescaled when stiffness is reduced [20]. In practice, the decision is usually made with a single rule: the mean overlap should stay below about 1% of the diameter, or the packing density should no longer change with stiffness.

These studies treat softening as a change of one property, the compliance of the contacts. In an explicit simulation it changes a second one as well. The time step is usually set as a fixed fraction of the Rayleigh time [5], which grows as the square root of the compliance, so a softened run differs from a stiff one both in how far its contacts deform and in how coarsely it is integrated. For the elastic contact forces, a fixed fraction keeps the integration error comparable between runs [6]. Other parts of the contact law, however, are not tied to the contact stiffness. A widely used rolling resistance model applies at every contact a torque of fixed magnitude that opposes the rotation of the grain; this type of model has no stiffness of its own and is known to behave poorly when a grain approaches rest [21]. In one step such a torque changes the angular velocity of a grain by an amount set by the time step alone, so in a softened run with a Rayleigh-based step, part of what appears as a stiffness effect may be a time step effect. A second question concerns the use of softened models for parametric studies, which depend less on absolute values than on differences: how much the friction changes when the sliding friction, the wall friction or the grain size is changed. Whether such differences survive softening has seldom been tested directly.

Here we address these questions in a ring shear cell filled with monodisperse glass beads. Annular shear simulations of monodisperse grains have previously been used by our group to examine the effects of normal stress, low confinement and shear rate, each at a single particle stiffness [22,23,24]; here, the stiffness itself is the variable. We consider monodisperse glass spheres with a constant torque rolling resistance, sheared in one ring shear cell at a normal stress of 10 kPa. The aim of this study is to determine how 18 macroscopic and micromechanical observables depend on the ratio of the particle shear modulus to the normal stress, *G*/σ_n_, between 10^3^ and 3 × 10^5^, how much of each dependence is caused by the time step rather than by the contact compliance, and whether the friction changes produced by five selected contact parameters are preserved when the particles are softened. To separate the time step from the compliance, three runs of the stiffness sweep were restarted with time steps up to eight times smaller than the Rayleigh-based one, and the rolling friction end member was run at three stiffnesses. The study introduces no new contact model or integration scheme; its contribution is to show that, with this widely used rolling resistance model, the friction loss of softened particles is a time step effect, whereas the changes of the contact network are genuine effects of the compliance. The comparisons are internal to the numerical model: they verify how the model output depends on the particle stiffness and the time step but do not validate the model against laboratory ring shear tests.

## 2. Materials and Methods

### 2.1. Ring Shear Model and Contact Law

**Figure 1:**
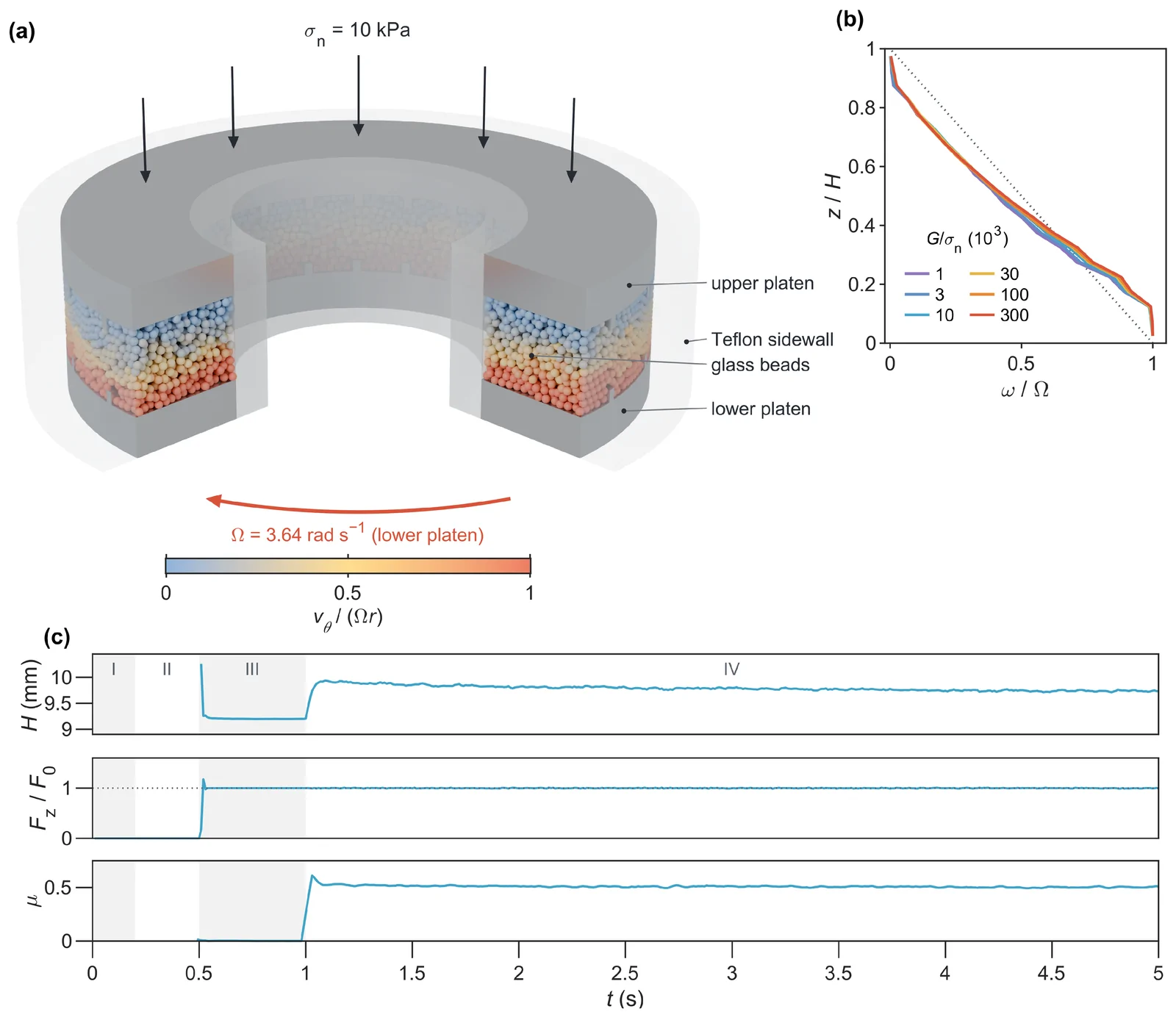
Ring shear model and loading. (**a**) Cut-away of the BASE bed (*G*/σ_n_ = 10^4^) at *t* = 3.5 s; grains are colored by their azimuthal velocity normalized by the local velocity of the lower platen, *v*_θ_/(Ω*r*). The lower platen rotates at Ω = 3.64 rad s^−1^, the upper platen is held at σ_n_ = 10 kPa without rotating, and both sidewalls are fixed. (**b**) Quasi-steady angular velocity profiles ω/Ω against normalized height *z*/*H* for the six stiffnesses; dotted line, linear profile. (**c**) Loading history of BASE: bed height *H*, normal force relative to its target *F*_z_/*F*_0_ and platen friction μ. Stages: I—grain generation; II—platen lowering; III—servo-controlled consolidation; IV—shear.

The simulated cell is an annulus with inner and outer radii *R*_i_ = 20 mm and *R*_o_ = 35 mm (Figure 1a). The bed is held between two toothed steel platens, each carrying 20 radial teeth (15 mm long, 1 mm wide and 1.5 mm high), and between fixed inner and outer sidewalls with the properties of Teflon. The full 360° annulus is modelled, without periodic boundaries. At the start of each run, a static generator placed glass beads of diameter *d* = 1 mm at random, non-overlapping positions in an annular region inside the cell (radii 22–33 mm, heights 4–46 mm). With a target mass of 0.038 kg and at most 20 placement attempts per grain, it placed 26,831–26,957 grains (0.0351–0.0353 kg) in the 1 mm runs and 3406–3423 grains (0.0357–0.0358 kg) in the 2 mm runs, so the bed mass differs by less than 2% between runs. The settled bed has a height *H* ≈ 9.8 mm, close to 10*d*. The lower platen rotates at Ω = 3.64 rad s^−1^, which moves the grains next to it at *v* = 0.1 m s^−1^ on the mid-radius circle (27.5 mm). The upper platen does not rotate; its vertical position is servo-controlled to hold a normal force *F*_z_ = 25.9 N, i.e., σ_n_ = 10 kPa on the annular area *A* = 2.59 × 10^−3^ m^2^. Gravity acts throughout, but the weight of the bed is only about 1.3% of the applied load.

The simulations were performed with LIGGGHTS-PUBLIC (version 3.8.0), an open source discrete element method package (https://github.com/CFDEMproject/LIGGGHTS-PUBLIC, accessed on 11 September 2026), using explicit first-order (Euler) time integration with a fixed time step. Grains interact through the Hertz–Mindlin no-slip law [2,3]. The elastic normal force between two spheres is

(1) $$F_{n}=\frac{4}{3}E^{*}\sqrt{R^{*}}\delta_{n}^{3/2}$$

where δ_n_ is the normal overlap, R* is the effective radius (*d*/4 for two equal grains) and E* the effective modulus, which for two grains of the same material with Young’s modulus *E* = 2*G*(1 + ν) and Poisson’s ratio ν reduces to *E*/[2(1 − ν^2^)]. A viscous force set by the coefficient of restitution e is added in the normal direction [25]:

(2) Fnd=−256βSnm*vn, β=ln eln2e+π2

where *S*_n_ = 2E*(R*δ_n_)^1/2^ is the normal contact stiffness, m* the effective mass (half the grain mass for two equal grains) and *v*_n_ the normal component of the relative velocity. The tangential force grows with the tangential displacement through the stiffness *S*_t_ = 8G*(R*δ_n_)^1/2^, with G* = *G*/[2(2 − ν)] for two equal grains [2,3]. It carries a damping term of the same form as Equation (2), with *S*_t_ in place of *S*_n_, and is limited by the Coulomb condition |**F**_t_| ≤ μ_s_*F*_n_. At fixed *e* the damping ratio β is fixed, so the energy lost in a binary impact does not depend on the stiffness, whereas the duration of the impact does. Hertz theory gives *t*_c_ ≈ 2.87(m*^2^/(R*E*^2^*v*_n_))^1/5^, which for an impact speed *v*_n_ = $\dot{\gamma }$*d* is about 1.7 × 10^−4^ s at *G*/σ_n_ = 10^3^, 7 × 10^−5^ s at 10^4^ and 1.8 × 10^−5^ s at 3 × 10^5^. Rolling resistance follows the standard constant-torque model, in which each contact exerts on a grain the torque

(3) $$\tau_{r}=-\mu_{r}F_{n}R_{c}\hat{\omega }$$

where *R*_c_ is the distance from the center of the grain to the contact point and $\hat{\omega }$ is the unit vector of the grain’s own angular velocity [21]. The magnitude of this torque is fixed by the normal force; only its direction follows the rotation, and it contains no rotational stiffness or damping. Contacts with the platens and the sidewalls use the same laws, with the effective modulus of the glass–steel or glass–Teflon pair, the wall treated as locally flat (R* = *d*/2) and the interaction coefficients of Table 1; rolling resistance also acts at these contacts. Table 1 lists all material and interaction parameters. Only the glass beads are softened; the platens and sidewalls keep their properties in every run.

### 2.2. Stiffness Sweep and End Member Checks

The particle shear modulus was varied from *G* = 0.01 to 3 GPa in six steps (runs K001, K003, BASE, K030, K100 and K300; Table 2), so that *G*/σ_n_ spans 10^3^–3 × 10^5^. For Hertzian contacts the distance from the rigid limit is measured by the stiffness number [13]

(4) $$\kappa ={\left(\frac{E}{\left(1-\nu^{2}\right)\sigma_{n}}\right)}^{2/3}$$

which ranges from 184 to 8255 here; the stiffest run corresponds to κ for real glass at a confining stress of about 100 kPa. A first estimate of the contact overlap follows from Equation (1) with the mean normal force ⟨*f*⟩ ≈ πσ_n_*d*^2^/(*Z*φ), where *Z* is the coordination number and φ the solid fraction:

(5) $$\frac{\delta_{n}}{d}\approx {\left(\frac{3\pi \sigma_{n}}{2\mathrm{Z\varphi}E^{*}}\right)}^{2/3}$$

End member runs (μ_s_ = 0.1, μ_r_ = 0, μ_w_ = 0.6, *d* = 2 mm, *e* = 0.1) were run at *G* = 0.1 GPa and repeated at 0.3 GPa; the μ_r_ = 0 end member was also run at 0.01 and 1 GPa, the *e* = 0.1 end member at 1 GPa, and the μ_r_ = 0 end member at 0.1 GPa was restarted with half its step.

**Figure 2:**
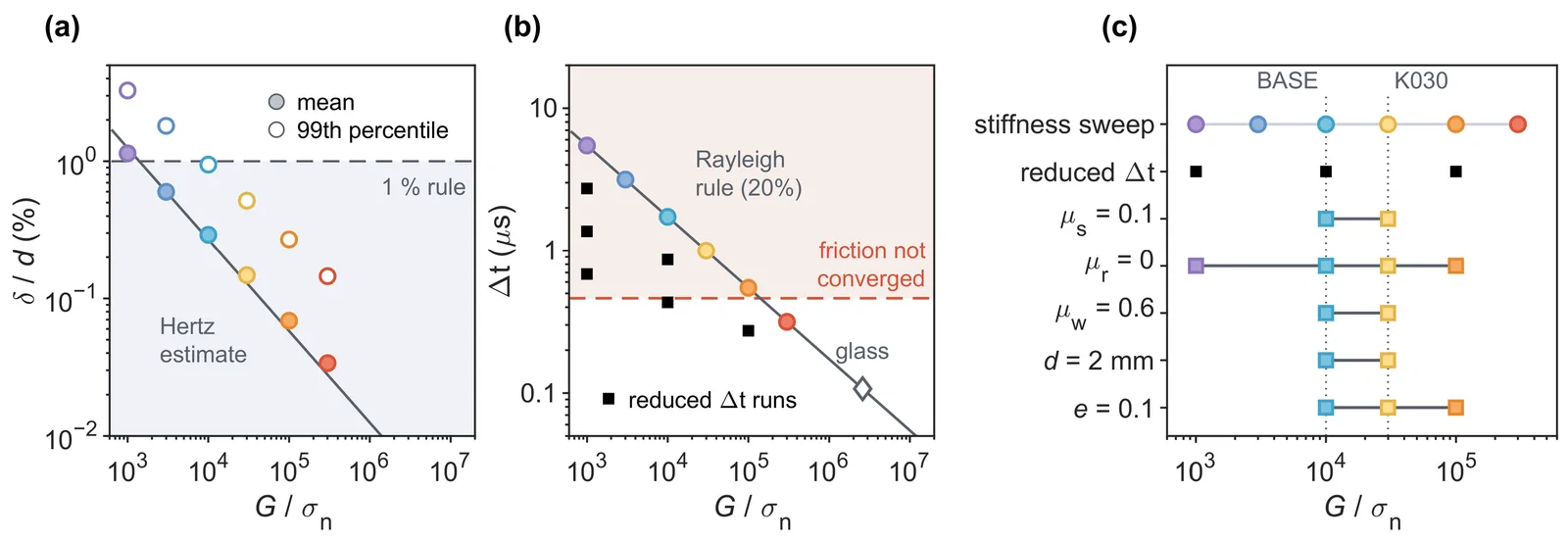
Design of the stiffness sweep and the time step tests. (**a**) Mean (filled) and 99th-percentile (open) contact overlap δ/*d* against *G*/σ_n_; line: Hertz estimate of Equation (5); dashed line: the 1% rule. (**b**) Time step against *G*/σ_n_: line and circles, 20% of the Rayleigh time (Equation (6)) used in the sweep; squares: reduced steps of the restarted runs; diamond: real glass (*G* ≈ 26 GPa) at 10 kPa; shaded above the dashed line: steps at which the friction lies more than 1% below its converged value (Ω_r_ > 0.7, Section 3.2). (**c**) Case matrix: the six-run stiffness sweep, the restarted runs with reduced time steps, and the five end-members, each run at *G*/σ_n_ = 10^4^ and 3 × 10^4^ and, for μ_r_ = 0 and *e* = 0.1, at further stiffnesses. Symbol colors in (**a**–**c**) give *G*/σ_n_: purple, 10^3^; medium blue, 3 × 10^3^; light blue, 10^4^; yellow, 3 × 10^4^; orange, 10^5^; red, 3 × 10^5^. The grey symbols in the legend of (**a**) show the marker style only.

With *Z* = 4.5 and φ = 0.6, Equation (5) predicts the measured mean overlaps to within 20% (Figure 2a). The mean overlap falls below 1% of *d* from *G*/σ_n_ = 3 × 10^3^ upwards, and its 99th percentile from 10^4^ upwards. In the sweep the time step was set, as is common practice, to the same fraction, 20%, of the Rayleigh time in every run. The choice of a stable increment is discussed in [5]; because integration errors grow superlinearly with the time step [6], a fixed fraction keeps the errors of the elastic contact forces comparable across the sweep. The Rayleigh-based step is

(6) $$\Delta t=\frac{0.2\pi R\sqrt{\frac{\rho }{G}}}{0.1631\nu +0.8766}$$

with *R* = *d*/2 and ρ the particle density, so it falls as (*G*/σ_n_)^−1/2^ across the sweep (Figure 2b) and the cost of a run with the same number of grains and the same simulated time grows as (*G*/σ_n_)^1/2^. The stiffest run costs 5.5 times as much as BASE, and real glass at 10 kPa would cost 16 times as much. Because the platens and sidewalls keep their properties, the ratio of wall to particle stiffness also falls as *G* increases; this is inherent in softening only the particles and is part of what the sweep tests.

To test whether softening preserves parametric trends, five end-members of the companion parameter studies were run at two stiffnesses, *G* = 0.1 and 0.3 GPa (*G*/σ_n_ = 10^4^ and 3 × 10^4^), and compared with BASE and K030, respectively (Figure 2c). Each end member changes one parameter from its BASE value: the glass–glass sliding friction (μ_s_ = 0.1), the rolling friction (μ_r_ = 0), the glass–sidewall friction (μ_w_ = 0.6), the grain diameter (*d* = 2 mm) or the restitution coefficient (*e* = 0.1). The rolling friction and restitution end members were also run at *G* = 1 GPa (*G*/σ_n_ = 10^5^) and compared with K100, and the rolling friction end member was run at *G* = 0.01 GPa (10^3^) as well. Doubling the grain size at the same bed mass also changes the confinement: the grain number falls to about 3400, the bed height drops from about 10 to 5 grain diameters, and the inertial number nearly doubles, to about 0.013. The grain mass and moment of inertia grow eightfold and 32-fold, and the cell is resolved by fewer grains. The *d* = 2 mm end member therefore tests the combined effect of grain size and finite cell size rather than the effect of grain size alone.

### 2.3. Loading Protocol and Comparison Window

Each run followed the same sequence (Figure 1c). The grains were placed at *t* ≈ 0 and settled under gravity until 0.2 s; every run, including the end members, generated its own random packing in this way. The upper platen (mass *m*_p_ = 0.105 kg) was then lowered at 0.1 m s^−1^ between 0.2 and 0.5 s. From 0.5 s onwards it is moved by a force controller that integrates its vertical equation of motion

(7) $$m_{p}{\ddot{z}}_{p}=F_{g,z}-F_{0},\;\left|{\dot{z}}_{p}\right|\leq 2\;{m\;s}^{-1}$$

where *z*_p_ is the platen height, *F*_g,z_ the vertical force exerted on the platen by the grains and *F*_0_ = 25.9 N the target load. The controller applies no additional damping, its speed is capped at 2 m s^−1^, gravity is not applied to the platen, and its other degrees of freedom are fixed. The bed was consolidated in this way between 0.5 and 1.0 s; averaged over the comparison window, the normal force stays within 0.2% of its target. The lower platen started rotating at 1.0 s, and shear continued to *t* = 4 s (5 s for BASE). Unless stated otherwise, results are averages over 3–4 s, after a nominal shear strain *vt*/*H* of about 20. We call this interval the comparison window and refer to averages over it as quasi-steady values, because the friction has not fully stopped evolving (see below and Appendix A). In every run, the angular velocity profile is close to linear across the whole bed, and the profiles of the six stiffnesses superpose (Figure 1b): there is no localized shear band and the slip at the platens is at most 0.3% of the imposed velocity. The nominal shear rate is *v*/*H* ≈ 10.2 s^−1^, which gives a nominal inertial number *I*_0_ = (*v*/*H*)*d*/(σ_n_/ρ)^1/2^ ≈ 5 × 10^−3^. Because the grains between the teeth move with the platens, the shear is carried by the central part of the bed; the measured rate $\dot{\gamma }$, taken as 80% of the velocity difference Ω$\bar{r}$ divided by the thickness of the layer in which the angular velocity drops from 0.9Ω to 0.1Ω, is about 14 s^−1^ and gives *I* ≈ 7 × 10^−3^. Both values lie at the slow end of the dense flow regime [14,26]; the measured $\dot{\gamma }$ is used to normalize the granular temperature, the diffusion coefficients and the rolling impulse of Section 2.5.

### 2.4. Observables and Uncertainties

The platen friction is the ratio of the torque *T* on the upper platen to the instantaneous normal force on it:

(8) $$\mu =\frac{T}{R_{\mathrm{eff}}F_{z}}$$

where *R*_eff_ = 2(*R*_o_^3^ − *R*_i_^3^)/[3(*R*_o_^2^ − *R*_i_^2^)] = 28.2 mm is the radius at which a uniform shear stress produces the measured torque. An independent estimate comes from the contact stress tensor σ_ij_ = *V*^−1^Σ*f*_i_*l*_j_, summed over grain–grain contacts with force **f** and branch vector **l** in the bed volume *V*, and written in cylindrical components: μ_σ_ = σ_θz_/σ_zz_. The solid fraction φ is the grain volume divided by *AH*; φ_0_ is its value just before shear, and the dilatancy Δ*H* is the increase in bed height between the start of shear and the averaging window.

The contact network is described by the coordination number *Z* = 2*N*_c_/*N* (grain–grain contacts *N*_c_, grains *N*), the rattler fraction (grains with fewer than two contacts, walls included), the coordination number *Z*_m_ of the non-rattlers, the mean and 99th-percentile overlap, and the contact persistence, i.e., the fraction of contacts present in one stored frame that still exist 0.01 s later. Sliding contacts are those with |**f**_t_| ≥ 0.98μ_s_*f*_n_; we report the share of the total normal force they carry. Force transmission is characterized by the distribution *P*(*f*/⟨*f*⟩) of normal contact forces, the exponent β of an exponential fit to its tail (1 < *f*/⟨*f*⟩ < 4) and the fraction of contacts that carry more than the mean force [27]. The anisotropies of contact orientation and normal force in the shear plane (θ, *z*) follow from the fabric tensor *R*_ij_ = ⟨*n*_i_*n*_j_⟩ of the contact normals **n** and its force-weighted counterpart *R*^f^_ij_ = ⟨(*f*_n_/⟨*f*_n_⟩)*n*_i_*n*_j_⟩:

(9) $$a_{c}=2\sqrt{{\left(\frac{R_{\theta \theta }-R_{\mathrm{zz}}}{2}\right)}^{2}+{R_{\mathrm{\theta z}}}^{2}},\;a_{n}=2\sqrt{{\left(\frac{R_{\theta \theta }^{f}-R_{\mathrm{zz}}^{f}}{2}\right)}^{2}+{\left(R_{\mathrm{\theta z}}^{f}\right)}^{2}}-a_{c}$$

Particle mobility is described by the granular temperature *T*_g_, one-third of the mean squared velocity fluctuation about the local mean velocity, normalized by ($\dot{\gamma }$*d*)^2^, and by the vertical and radial self-diffusion coefficients *D*_z_ and *D*_r_, obtained from the slope of the mean squared displacement of grains in the middle 40% of the bed over 0.1–0.5 s and normalized by $\dot{\gamma }$*d*^2^. The spin fluctuation is the standard deviation, over all grains, of the radial component of the angular velocity, the component about which the mean shear rotates the grains. Crystalline order is measured with the locally averaged bond-order parameter $\bar{Q}$_6_ [28,29], with neighbors within 1.25*d* and grains with $\bar{Q}$_6_ > 0.4 counted as crystalline. Finally, the sidewall share is the torque taken up by the two sidewalls divided by the driving torque on the lower platen.

Within the comparison window the friction still drifts slowly in some runs. The linear trend over the window is at most 2.3% of the mean in the stiffness sweep, but it reaches 4–6% in the end members without rolling friction and in the low-sliding friction run at the higher stiffness. Sheared packings of monodisperse spheres are known to crystallize [30,31], and BASE continued to 10 s shows that its friction falls by 2.5% between the 3–4 s window and 9–10 s as ordered layers grow at both platens, while the mid-bed structure does not change (Appendix A). Because each end member drifts largely in step with its reference run, the friction changes used in Section 3.7 are insensitive to the choice of window: evaluating them over 3–3.5 s or 3.5–4 s instead of 3–4 s changes the difference between the two stiffnesses by at most 0.003, except for the 2 mm grains, where it ranges from −0.014 to +0.003.

Three sources of uncertainty are distinguished. (i) Temporal fluctuations: the comparison window holds 101 frames 0.01 s apart, and the integrated autocorrelation time of the platen friction, computed after removing the linear drift, is 0.005–0.009 s, shorter than the sampling interval. Five consecutive blocks of 0.2 s, each about 25 autocorrelation times long, are therefore effectively independent, and the block standard error of the platen friction is 0.0005–0.002 for runs without drift (up to 0.005 for the 2 mm grains, which number eight times fewer). Using between 5 and 20 blocks changes it by less than a factor of two (Appendix B). (ii) Drift: in runs with a drift of 4–6%, the block error grows to 0.002–0.003 because the drift is counted as fluctuation; after removing the linear trend it falls to 0.0003–0.0007. The drift is reported separately rather than folded into the error bars. (iii) Packing-to-packing variability: BASE and the rolling friction end member at *G*/σ_n_ = 10^4^ were each repeated with two further independently generated packings, and K030 with one. The standard deviation of the platen friction between packings is 0.0007 for BASE and 0.0023 for K030, which gives about 0.002 for the difference between two runs with rolling resistance, and 0.010 for the end member without rolling resistance, whose packings crystallize at different rates (Appendix B, Table A1). Grey bands of ±3% around the K300 value are drawn in the figures as a working tolerance for reading the stiffness dependence; the tolerance is larger than the statistical uncertainty of the friction and comparable to the 2.5% long-time drift of BASE.

### 2.5. Time Step Tests

To separate the time step from the contact compliance, K001, BASE and K100 were restarted from their stored state at *t* = 2.0 s with smaller time steps (Figure 2b,c): K001 with 1/2, 1/4 and 1/8 of its Rayleigh-based step, BASE with 1/2 and 1/4, and K100 with 1/2. The rolling friction end member at 10^4^ was restarted in the same way with half its step. Only the time step was changed; the packing and the shear history up to 2.0 s are those of the original runs. The restarted runs were continued to 3 s (the half-step runs of K001 and K100 to 4 s), and because the friction responded to a new time step within one stored frame (Section 3.2), restarted runs and their originals are compared over 2.2–3.0 s.

The time step enters the rolling resistance directly. All contact torques of Equation (3) on one grain act along −$\hat{\omega }$, so they add up to μ_r_*R*Σ*F*_n_, and in one time step they change the angular velocity of the grain by Δω_r_ = μ_r_*R*Σ*F*_n_Δ*t*/*J*, with the moment of inertia *J* = πρ*d*^5^/60. With the sum of the normal forces on a grain estimated as Σ*F*_n_ ≈ πσ_n_*d*^2^/φ, as in Equation (5), this per-step angular impulse becomes

(10) $${\Delta \omega }_{r}\approx \frac{30\mu_{r}\sigma_{n}\Delta t}{\varphi \rho d^{2}},\;\Omega_{r}=\frac{{\Delta \omega }_{r}}{\dot{\gamma }}$$

and its ratio to the shear rate compares it with the rotation rates imposed by the flow. With Rayleigh-based steps, Ω_r_ falls from 7.9 in K001 to 0.47 in K300; the restarted runs reach 0.41.

For each stiffness with a restarted run, the deviation of an observable *X* from K300 over 2.2–3.0 s is split into a time step part, *X*(Rayleigh step) − *X*(smallest step), and a compliance part, *X*(smallest step) − *X*(K300), both expressed as a percentage of the K300 value. K300 can serve as the reference because its friction agrees within 0.1% with that of the restarted runs at the smallest steps (Section 3.2). In Section 3, the time step-corrected values are the 3–4 s values of the original runs plus the change measured over 2.2–3.0 s on reducing the time step.

## 3. Results

### 3.1. With Rayleigh-Based Time Steps, Softer Grains Carry Lower Friction

**Figure 3:**
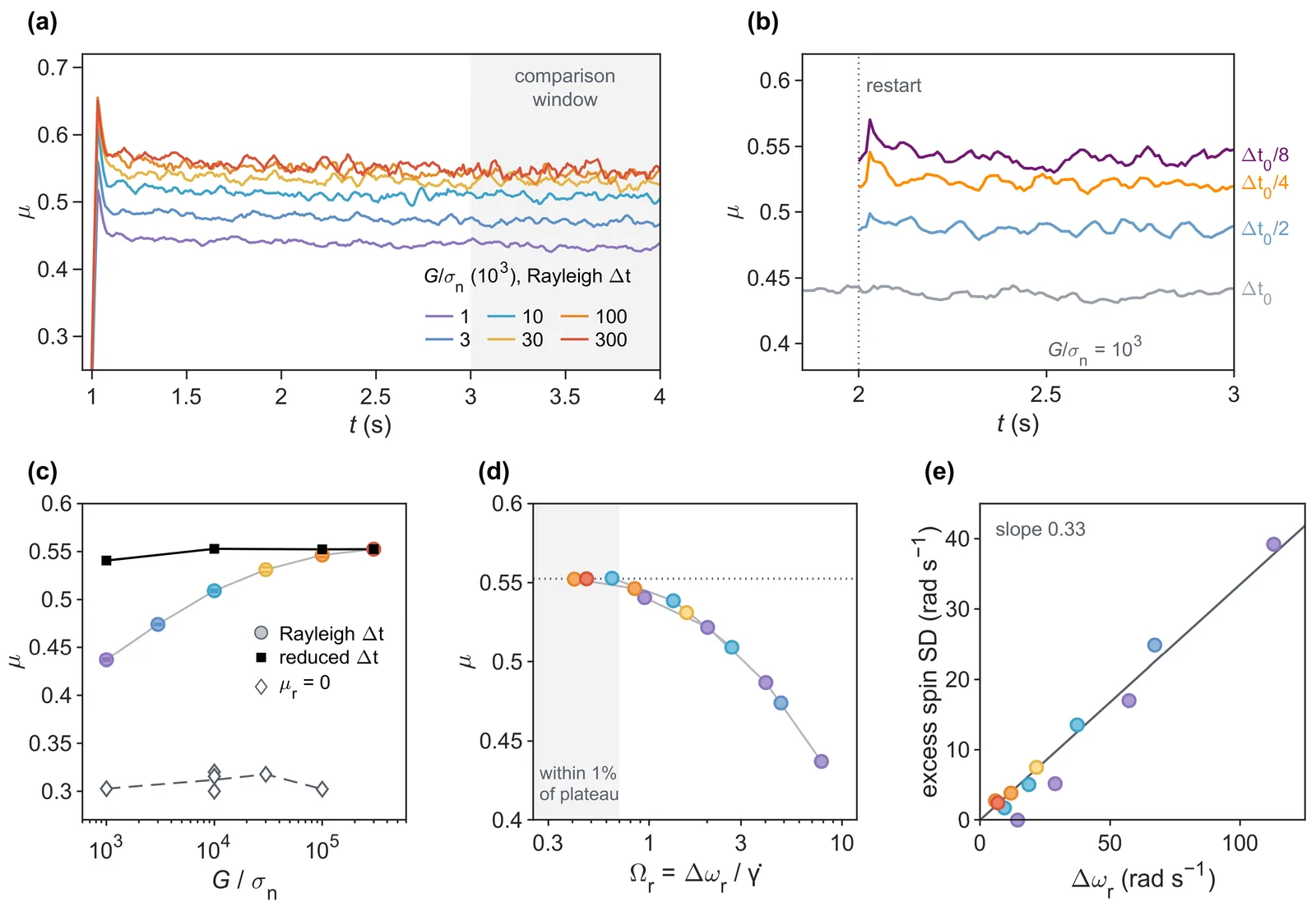
Friction and time step. (**a**) Platen friction μ(*t*) for the six stiffnesses with Rayleigh-based time steps (0.05 s running mean); shaded, comparison window (3–4 s). (**b**) K001 (*G*/σ_n_ = 10^3^) restarted at *t* = 2.0 s with 1/2, 1/4 and 1/8 of its time step Δ*t*_0_. (**c**) Friction over 2.2–3.0 s against *G*/σ_n_ with Rayleigh-based steps (circles; error bars, block-averaged standard errors) and at the smallest steps run (squares), and friction without rolling resistance over 3–4 s (diamonds; three packings at 10^4^). (**d**) Friction of all runs with rolling resistance against the per-step impulse ratio Ω_r_ of Equation (10); colors give *G*/σ_n_ as in (**a**); lines join runs of the same stiffness; dotted line: plateau at the smallest steps; shaded: within 1% of the plateau. (**e**) Excess spin fluctuation: the standard deviation of the radial angular velocity above its converged value of 16.6 rad s^−1^, against the per-step angular impulse Δω_r_; line: fit through the origin. Symbol colors in (**c**–**e**) give *G*/σ_n_ as in (**a**). In (**a**), the curves for *G*/σ_n_ ≥ 3 × 10^4^ lie close together because their friction differs by only a few percent; in (b), each curve is labelled by its time step.

The friction history has the same shape at every stiffness (Figure 3a). It jumps to a peak about 0.03 s after the platen starts rotating, relaxes within roughly one nominal unit of strain, and then fluctuates about a quasi-steady value. This comparison-window average, however, rises steadily with stiffness, from 0.434 at *G*/σ_n_ = 10^3^ to 0.547 at 3 × 10^5^, an increase of 26%. The contact stress estimate rises in step, from 0.389 to 0.492. It is lower than the platen value because it excludes the grain–platen contacts at the teeth, but its relative change with stiffness is almost the same. Relative to K300, the platen friction is 7.0% lower at BASE (10^4^), 3.0% lower at 3 × 10^4^ and 1.2% lower at 10^5^. Read in the usual way, this sweep would say that the commonly used ratio of 10^4^ underestimates the shear strength by about 7% and that the friction approaches its stiff value only slowly. The next section shows that this reading does not hold.

### 3.2. The Friction Loss Is a Time Step Effect

When K001 was restarted at 2.0 s with half its time step, its friction rose from 0.437 to 0.487 within the first stored frame and stayed there (Figure 3b). A quarter and an eighth of the step raised it further, to 0.522 and 0.541, all averaged over 2.2–3.0 s. The packing had no time to change: the restarted runs began from the same grain positions, and the response was complete before any appreciable strain had accumulated. BASE behaved in the same way, rising from 0.509 to 0.539 at half and 0.553 at a quarter of its step, whereas K100 rose by only 1.1%, from 0.546 to 0.552.

Plotted against the stiffness, the friction of the restarted runs is nearly flat (Figure 3c). At the smallest steps, BASE (0.553), K100 (0.552) and K300 (0.552) agree to within 0.001, and K001 (0.541) lies 2.1% lower and was still rising, by 3.6% at the last halving of its step. A stiffness ratio of 10^4^ therefore reproduces the friction of the stiffest grains within the numerical uncertainty once its time step is small enough, and the 26% friction loss of Section 3.1 is almost entirely a consequence of the larger time steps of the softer runs. When all twelve runs with rolling resistance are plotted against the per-step impulse ratio Ω_r_ of Equation (10), they fall on one curve irrespective of their stiffness (Figure 3d). For example, K001 at a quarter of its step (Δ*t* = 1.37 µs) has the friction that BASE has at the same time step, 0.522 against 0.521 interpolated linearly in Δ*t* between the BASE runs. The friction lies within 1% of the plateau of 0.552 for Ω_r_ ≤ 0.64 and falls increasingly below it at larger values: by 1.1% at Ω_r_ = 0.84 (K100), 7.8% at 2.7 (BASE) and 21% at 7.9 (K001).

The runs without rolling resistance confirm that the time step acts through the rolling torque. Halving the step of the μ_r_ = 0 end member changed its friction by 0.5% (0.331 against 0.329), and across the stiffness range the friction of this end member shows no trend—0.303 at 10^3^, 0.300–0.320 for three packings at 10^4^, 0.318 at 3 × 10^4^ and 0.302 at 10^5^ (Figure 3c)—although its coordination number falls from 4.83 to 3.65 over the same range. Without rolling resistance, the friction depends neither on the time step nor on the stiffness.

The rolling torque leaves a direct trace in the rotation of the grains. At the smallest steps the spin fluctuation is 16.6 rad s^−1^ on average and independent of stiffness. With Rayleigh-based steps it grows to 21.4 rad s^−1^ in BASE and 42.6 rad s^−1^ in K001, and the excess over the converged value increases in proportion to the per-step impulse Δω_r_, with a slope of 0.33 (Figure 3e). Without rolling resistance the spin fluctuation is 54 rad s^−1^. A large time step thus makes the grains spin more freely, as if part of their rolling resistance had been removed.

### 3.3. Packing, Dilatancy and Diffusion

**Figure 4:**
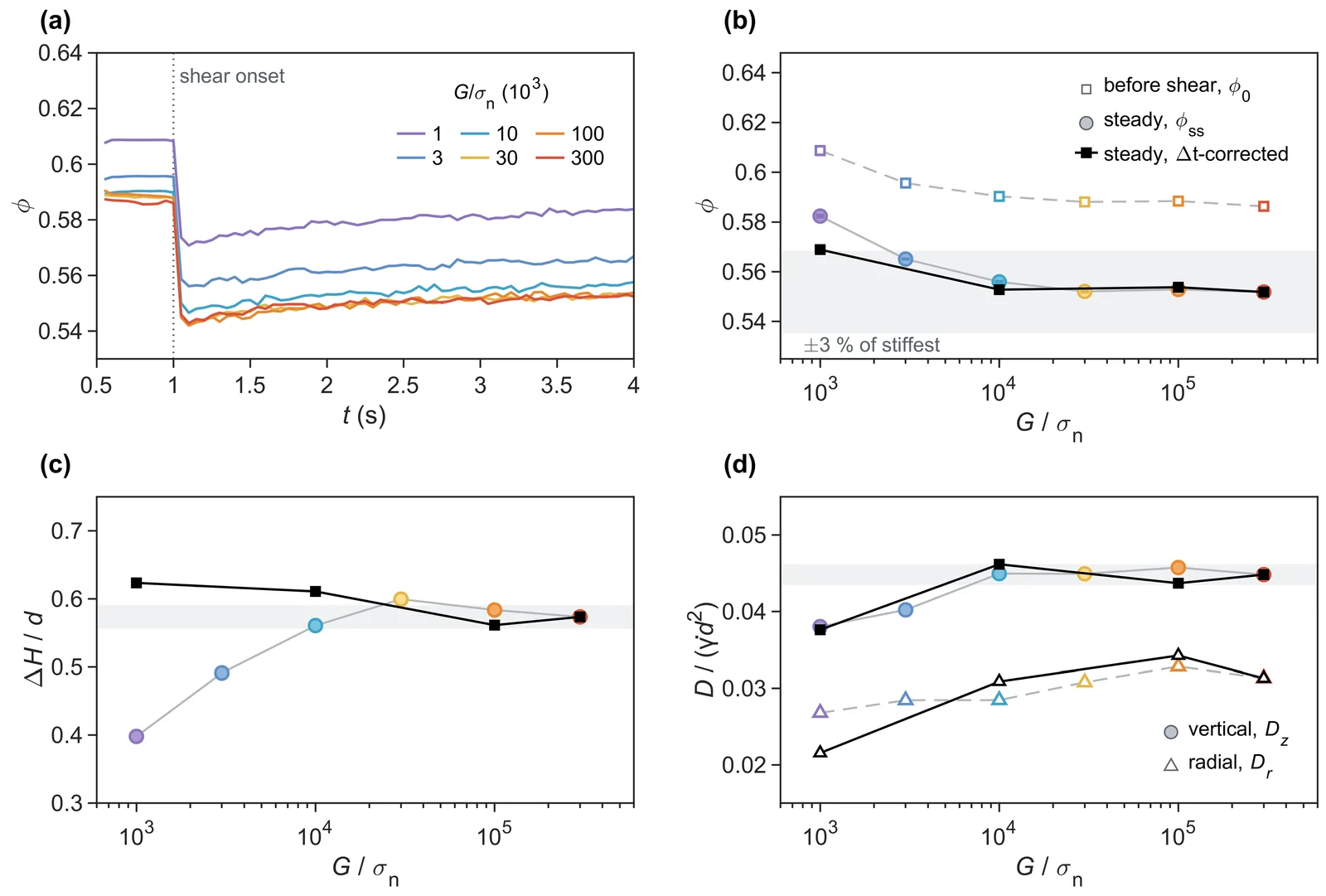
Packing, dilatancy and diffusion. (**a**) Solid fraction φ(*t*); dotted line: onset of shear. (**b**) Quasi-steady solid fraction φ_ss_ (circles) and pre-shear value φ_0_ (squares). (**c**) Dilatancy Δ*H*/*d* between the onset of shear and the comparison window. (**d**) Vertical (circles) and radial (triangles) self-diffusion coefficients normalized by $\dot{\gamma }$*d*^2^. Colored symbols: Rayleigh-based time steps; black symbols: time step-corrected values (Section 2.5); grey bands: ±3% of the stiffest run. In (**a**), the curves for *G*/σ_n_ ≥ 10^4^ lie close together because their solid fractions differ by less than 1%.

The solid fraction falls with stiffness (Figure 4a,b). All beds dilate abruptly at the onset of shear and then compact slowly as ordered layers grow near the platens. With Rayleigh-based steps, the quasi-steady solid fraction falls from φ = 0.582 at *G*/σ_n_ = 10^3^ to 0.552 at 3 × 10^5^, and almost the whole change occurs below 10^4^. The pre-shear packing follows the same course, from 0.609 to 0.586. Part of this trend is numerical: at 10^3^, the time step accounts for 2.4 percentage points of the 5.4% excess over K300, and at 10^4^ the solid fraction agrees with K300 within 0.1% once the step is reduced (black symbols in Figure 4b). The remaining excess of the softest grains is a compliance effect. An affine estimate, in which the bed is compressed by the mean overlap in all three directions, gives Δφ ≈ 3φ⟨δ⟩/*d* ≈ 0.019 between the softest and the stiffest grains, close to the compliance part of 0.016.

The dilatancy is almost entirely a time step effect (Figure 4c). With Rayleigh-based steps, soft beds expand less on shearing, Δ*H*/*d* = 0.40 at 10^3^ against 0.56–0.60 from 10^4^ upwards. With reduced steps, the expansion of the soft beds is no longer smaller but slightly larger than that of K300 (time step-corrected values of 0.62 at 10^3^ and 0.61 at 10^4^, against 0.57 for K300). Vertical diffusion is hardly affected by the time step (at most 5%) and rises with stiffness from *D*_z_/($\dot{\gamma }$*d*^2^) = 0.038 to 0.045, agreeing with K300 within 3% from 10^4^ (Figure 4d). The radial coefficient is about two-thirds of the vertical one and noisier, because radial excursions are limited by the sidewalls. Because K100 lies 5% above K300, it does not meet the ±3% criterion within the sweep, and both its time step and its compliance parts reach 15–25% at 10^3^.

### 3.4. The Contact Network Changes with Stiffness

**Figure 5:**
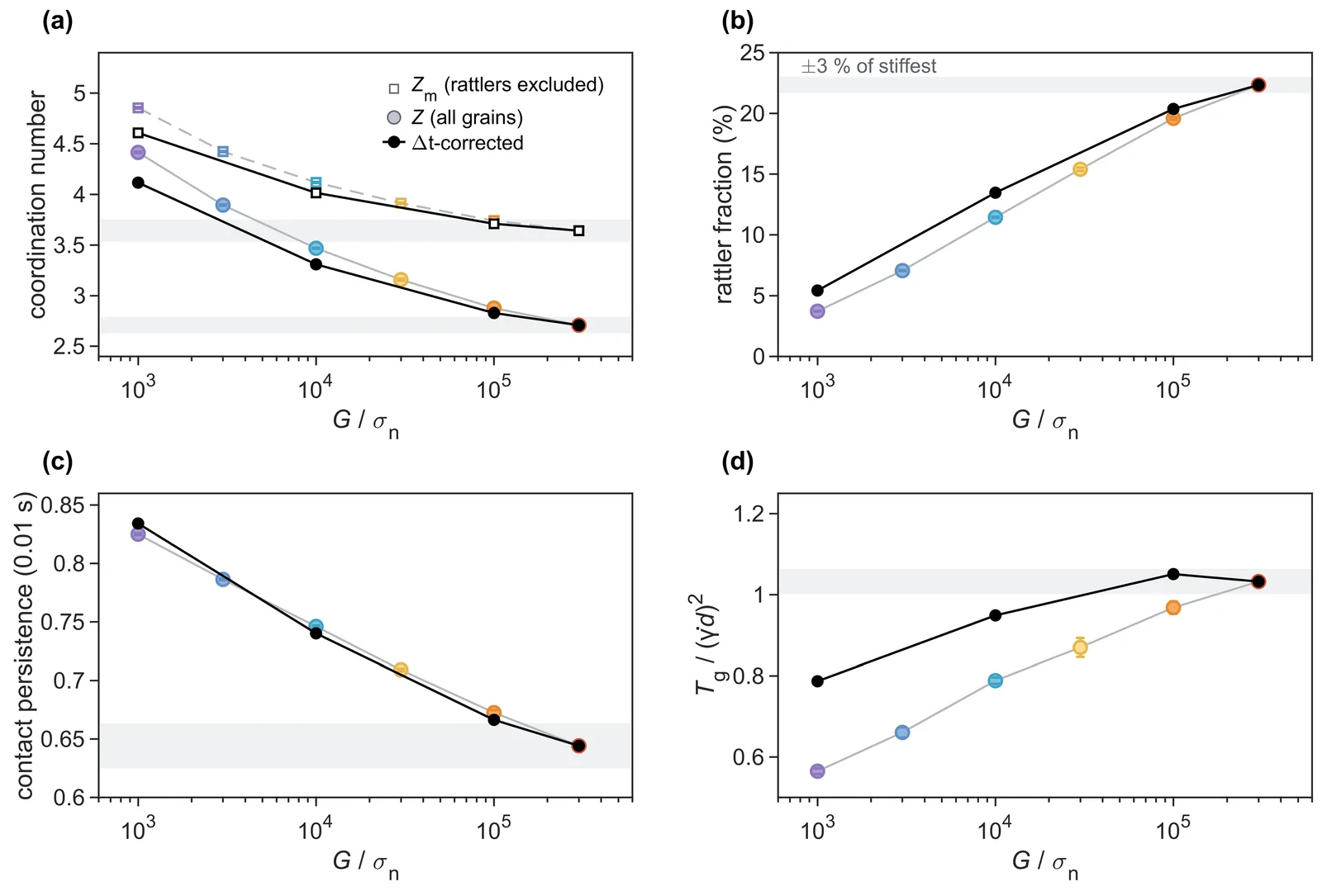
Contact network. (**a**) Coordination number of all grains, *Z* (circles), and of non-rattlers, *Z*_m_ (squares). (**b**) Rattler fraction. (**c**) Contact persistence: the fraction of contacts that survive one stored interval of 0.01 s. (**d**) Granular temperature *T*_g_/($\dot{\gamma }$*d*)^2^. Colored symbols: Rayleigh-based time steps (error bars, block-averaged standard errors); black symbols: time step-corrected values (Section 2.5); grey bands: ±3% of the stiffest run.

Quantities that count contacts change strongly with stiffness (Figure 5). With Rayleigh-based steps, the coordination number falls from *Z* = 4.41 at *G*/σ_n_ = 10^3^ to 2.71 at 3 × 10^5^, and *Z*_m_, which excludes rattlers, falls from 4.85 to 3.64 (Figure 5a). The rattler fraction rises sixfold, from 3.7% to 22.3% (Figure 5b). Contacts become shorter-lived as the grains stiffen: the fraction of contacts that survive 0.01 s drops from 0.83 to 0.64 (Figure 5c). The granular temperature almost doubles, from 0.56 to 1.03 in units of ($\dot{\gamma }$*d*)^2^ (Figure 5d). Unlike the friction, the network statistics keep most of these trends when the time step is reduced (black symbols). At 10^3^, the coordination number of the restarted run is still 51% above K300, the rattler fraction 75% below it and the contact persistence 29% above it; at 10^4^, the corresponding compliance parts are 22%, −39% and 14%. The time step adds smaller contributions in the same direction, 11% and 6% to *Z*. Even at 10^5^, *Z* is 5% above and the rattler fraction 10% below K300. The granular temperature is the exception in this group: about half of its trend is numerical, with time step parts of −21% and −15% at 10^3^ and 10^4^ against compliance parts of −23% and −9%.

These trends expose the weakness of the overlap rule. At *G*/σ_n_ = 3 × 10^3^, where the mean overlap is only 0.6% of *d*, the solid fraction is already within 2.4% of the stiffest run. At the same stiffness, however, the coordination number is 44% high and the rattler fraction 68% low, and, judging from the restarted runs at 10^3^ and 10^4^, most of these differences come from the compliance rather than from the time step.

### 3.5. Force Transmission and Fabric

**Figure 6:**
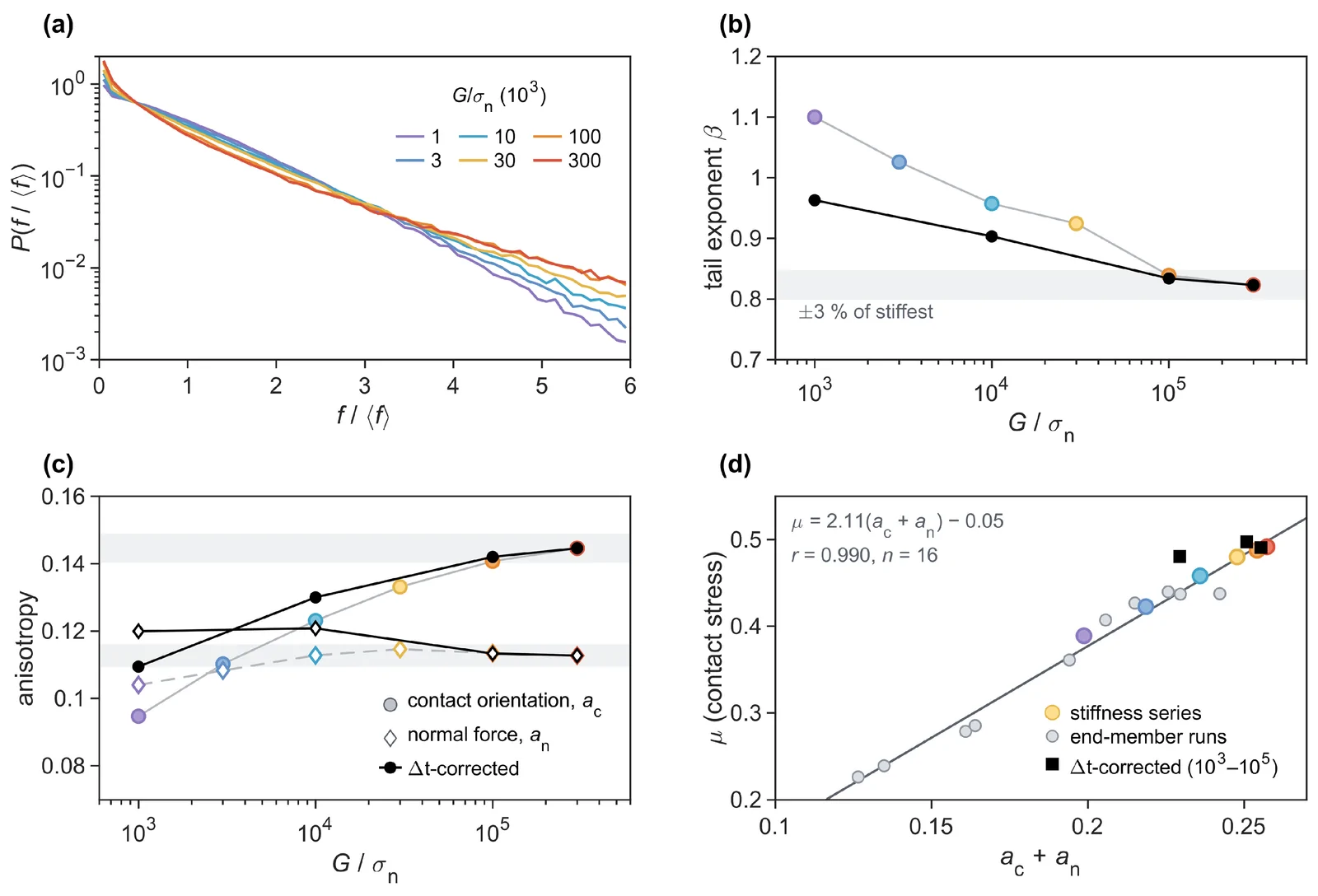
Force transmission and fabric. (**a**) Distribution of normal contact forces *P*(*f*/⟨*f*⟩) for the six stiffnesses. (**b**) Exponent β of the exponential tail (1 < *f*/⟨*f*⟩ < 4). (**c**) Contact orientation anisotropy *a*_c_ (circles) and normal force anisotropy *a*_n_ (diamonds) in the shear plane. (**d**) Contact stress friction against *a*_c_ + *a*_n_ for the 16 runs with Rayleigh-based time steps (stiffness sweep in color, end member runs at *G*/σ_n_ = 10^4^ and 3 × 10^4^ in grey; line: linear fit) and for the time step-corrected stiffness series at 10^3^, 10^4^ and 10^5^ (black squares). Black symbols in (**b**,**c**), time step-corrected values (Section 2.5). Line and symbol colors give *G*/σ_n_ as in the legend of (**a**); in (**d**), the legend symbol of the stiffness series shows one representative color. In (**a**), the six distributions overlap for *f*/⟨*f*⟩ < 3 and separate in the tail.

The contact forces also respond to stiffness. In stiffer beds the distribution of normal forces broadens: more contacts carry very small forces, and the tail of large forces extends further (Figure 6a). With Rayleigh-based steps, the exponent of the exponential tail decreases from β = 1.10 to 0.82 (Figure 6b), and the fraction of contacts carrying more than the mean force drops from 39% to 30%. The contact orientation anisotropy in the shear plane grows from *a*_c_ = 0.095 to 0.145 with stiffness, whereas the force anisotropy *a*_n_ varies only between 0.104 and 0.115 (Figure 6c). Reducing the time step moves both statistics of the force distribution and both anisotropies, but it does not remove their stiffness dependence: at 10^3^, the compliance parts are 22% for β, 23% for the strong contact fraction and −24% for *a*_c_, against time step parts of 17%, 9% and −10%. The force anisotropy is the only quantity of this group whose two parts have opposite signs: the time step lowers it (by 14% at 10^3^) and the compliance raises it (by 8%).

With Rayleigh-based steps, the contact stress friction of all 16 runs of this study, the six stiffnesses and the ten end member runs, is a single linear function of the total anisotropy, μ_σ_ = 2.11(*a*_c_ + *a*_n_) − 0.05 with *r* = 0.990 (Figure 6d), in line with the stress–force–fabric relation [32,33] and with earlier soft particle simulations [17]. The time step-corrected stiffness series does not follow this line. At 10^3^, the corrected friction (0.481) lies 0.04 above the value that the line predicts for its anisotropy, and at 10^4^ it lies 0.013 above it; only at 10^5^ does it fall on the line. Softer grains at a converged time step thus carry nearly the same friction with a less anisotropic contact orientation. Our anisotropies omit the tangential force contribution to the stress, which presumably makes up the difference; the single line of Figure 6d holds because, in the original sweep, the time step and the stiffness changed together.

### 3.6. Two Channels

**Figure 7:**
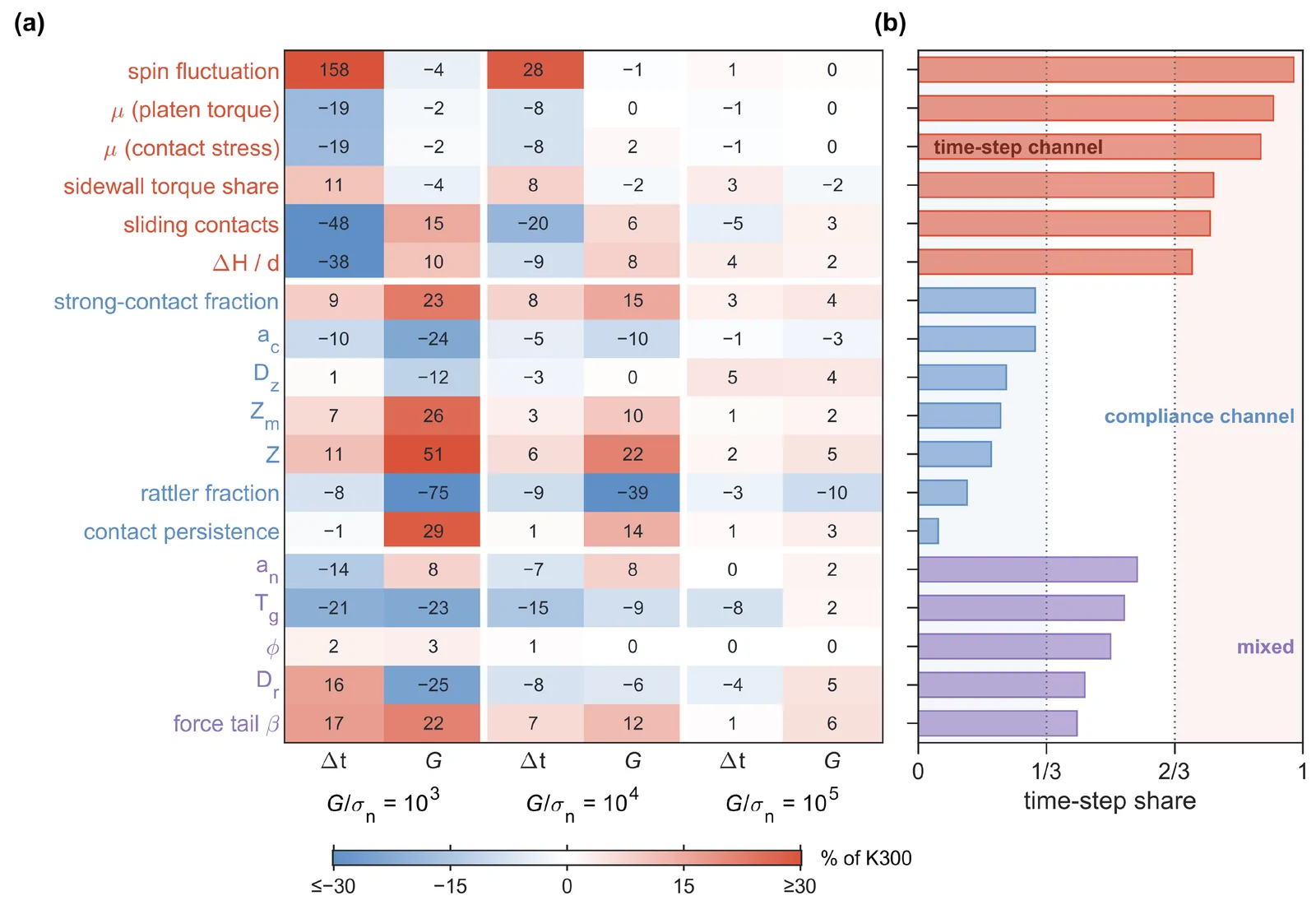
Time step and compliance parts of the stiffness effects. (**a**) Deviation of 18 observables from K300 at *G*/σ_n_ = 10^3^, 10^4^ and 10^5^, split into a time step part (Δ*t*: Rayleigh-based step minus smallest step) and a compliance part (*G*: smallest step minus K300), in % of the K300 value, over 2.2–3.0 s. (**b**) Share of the time-step part in the summed absolute deviations at 10^3^ and 10^4^; bar and label colors give the resulting group: time step channel (share > 2/3), compliance channel (<1/3) and mixed.

Figure 7 and Table 3 summarize the split of every stiffness effect into its time step and compliance parts. The observables fall into three groups (Figure 7b). In the first, the time step causes more than two-thirds of the deviation from K300 at 10^3^ and 10^4^: the platen and contact stress friction, the force share of sliding contacts, the sidewall torque share, the dilatancy and the spin fluctuation. These are the quantities that are tied to the rolling and sliding of grains. In the second, the compliance causes more than two-thirds: the coordination numbers, the rattler fraction, the contact persistence, the strong contact fraction, the contact anisotropy and the vertical diffusion. These are the quantities that count or orient contacts. The solid fraction, the force anisotropy, the granular temperature, the force tail exponent and the radial diffusion form a mixed group. At 10^5^, both parts are within about 5% for most observables; the largest are the compliance part of the rattler fraction (−10%) and the time step part of the granular temperature (−8%).

The split also changes the cost argument for softening. For the friction, the time step must be small enough that Ω_r_ ≲ 0.7, which in this cell means Δ*t* ≲ 0.45 µs. This is shorter than the Rayleigh-based step of every run softer than about *G*/σ_n_ = 1.4 × 10^5^ (Figure 2b). Below that stiffness, softening the grains no longer shortens a run whose friction must be converged, because the time step is then set by the rolling resistance rather than by the contacts.

### 3.7. Effects of Contact Parameters

**Figure 8:**
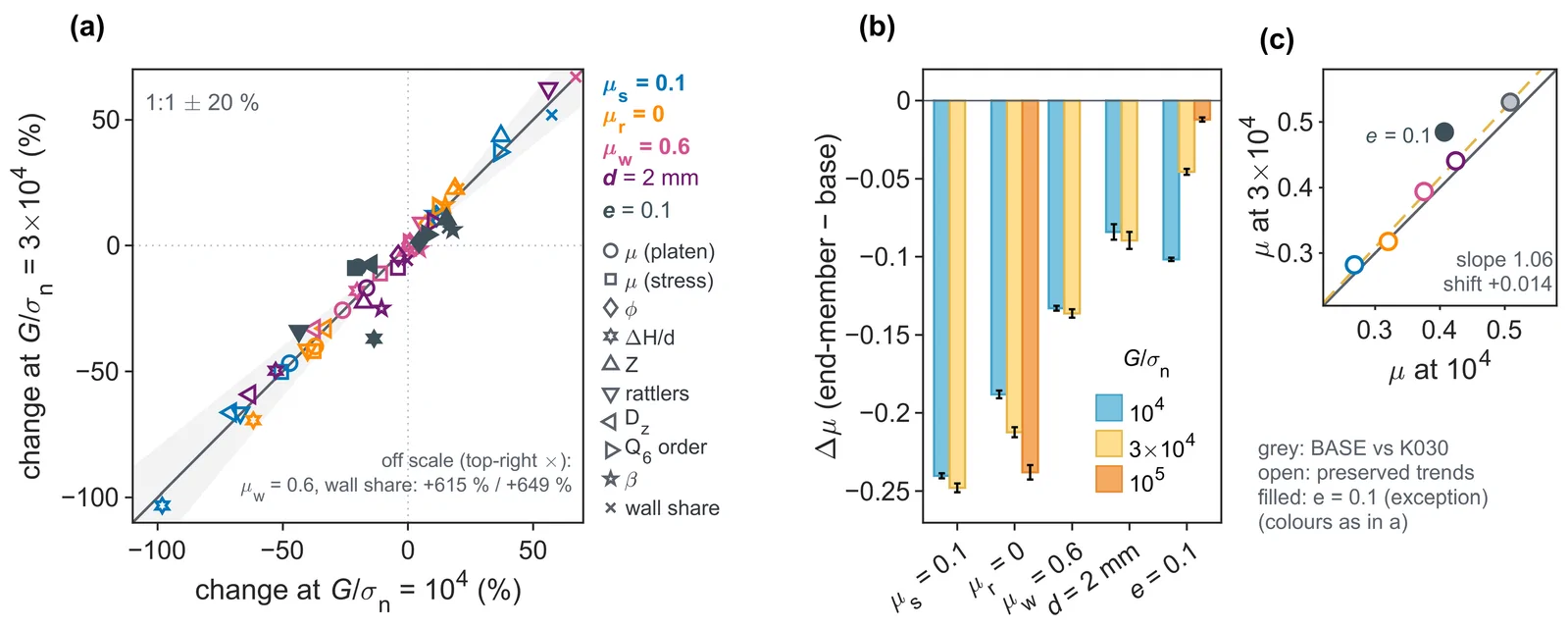
Robustness of parametric trends (Rayleigh-based time steps). (**a**) Relative change produced by each end member (color) in ten observables (symbols) at *G*/σ_n_ = 10^4^ (relative to BASE) and at 3 × 10^4^ (relative to K030); line—1:1; shaded—±20%; filled symbols—*e* = 0.1. (**b**) Friction change Δμ at 10^4^ and 3 × 10^4^ and, for μ_r_ = 0 and *e* = 0.1, at 10^5^ (relative to K100); error bars: combined block-averaged standard errors. (**c**) Absolute platen friction at 3 × 10^4^ against that at 10^4^: BASE and K030 (grey), the four preserved end-members (open) and *e* = 0.1 (filled); solid line—1:1; dashed line: fit through BASE and the preserved end-members.

The last test asks whether the effect of changing a contact parameter survives softening. Figure 8a compares the relative change that each end member produces in ten observables at *G*/σ_n_ = 10^4^ (relative to BASE) with the change it produces at 3 × 10^4^ (relative to K030); both pairs were run with their Rayleigh-based steps. For the four frictional and geometric parameters (μ_s_, μ_r_, μ_w_ and *d*), the points fall on the 1:1 line over two orders of magnitude of effect size, from a 0.7% change in φ to a sevenfold increase in the sidewall share at μ_w_ = 0.6. Of the 40 points, 37 lie within ±20% of the line or within 5 percentage points of it. The three exceptions are the force tail exponent β for the 2 mm grains and for μ_w_ = 0.6 and the small change in sidewall share for the 2 mm grains.

The friction changes themselves agree closely (Figure 8b). Lowering the sliding friction to 0.1 reduces μ by 0.240 at 10^4^ and by 0.248 at 3 × 10^4^. Raising the sidewall friction to 0.6 reduces it by 0.133 and 0.136, and doubling the grain size reduces it by 0.084 and 0.090. These differences of 0.003–0.008 are at most 2.4 times the combined block errors (0.003–0.007), comparable to the packing-to-packing uncertainty of a friction change (about 0.002 for runs with rolling resistance), and more than ten times smaller than the effects themselves. Because the base run and these end members all carry rolling resistance, the time step deficit of Section 3.2 lowers both members of each pair, and the differences are preserved. Figure 8c shows this directly: for BASE and the four preserved end members, the friction at 3 × 10^4^ follows that at 10^4^ along a line of slope 1.06, with a mean upward shift of 0.014.

The rolling friction end member is different, because only its reference run is affected by the time step. Removing rolling friction reduces μ by 0.188 at 10^4^, 0.212 at 3 × 10^4^ and 0.238 at 10^5^ (Figure 8b); the mean over three packings at 10^4^ is 0.196 ± 0.006. Since the friction without rolling resistance depends on neither the time step nor the stiffness (Section 3.2), this growth reflects the time step deficit of the reference runs, and the converged effect is close to the value at 10^5^, about −0.24. A softened model with a Rayleigh-based step therefore underestimates the strengthening by rolling resistance, which acts by hindering grain rotation [34], by about 20% at 10^4^. The restitution end member behaves differently again. Lowering *e* from 0.9 to 0.1 reduces μ by 0.102 at 10^4^, by 0.046 at 3 × 10^4^ and by only 0.012 at 10^5^, and its effects on the solid fraction, *Z*, β and the sidewall share shrink as well (filled symbols in Figure 8a). Because the stiffness and the time step changed together in these runs, the present data do not show which of the two removes the restitution effect. These statements are limited to one end member value per parameter, to the stiffness levels tested, and to this cell, stress and material.

## 4. Discussion

### 4.1. Why the Rolling Torque Makes Friction Depend on the Time Step

The constant torque model of Equation (3) prescribes the magnitude of the rolling torque and lets only its direction follow the rotation. When a grain rotates slowly, one step of this torque can more than cancel its angular velocity, and the next step, now acting in the opposite direction, reverses it again. The grain then chatters about zero rotation, and the torque, which alternates in sign, no longer resists the slow rolling that the flow imposes. This is the behavior that makes constant torque models problematic near rest [21], and in a sheared bed, where most grains rotate slowly and reverse often, it acts throughout the flow. The amplitude of the chatter is set by the angular impulse of one step, Δω_r_ of Equation (10), and the measured excess spin fluctuation grows in proportion to it (Figure 3e). The friction then lies between the values with and without rolling resistance: K001 with its Rayleigh-based step recovers only 54% of the friction that rolling resistance adds at a converged step (0.437, against 0.303 without rolling resistance over 3–4 s and 0.552 with rolling resistance at a converged step).

The stiffness enters this mechanism only through the time step rule. With the Rayleigh-based step of Equation (6), Equation (10) gives Ω_r_ ≈ 10.4(μ_r_/φ)(*G*/σ_n_)^−1/2^*I*^−1^, where *I* is the inertial number of Section 2.3, so the impulse ratio grows as the particles are softened and also as the inertial number falls. The latter dependence suggests that slow, quasi-static shear with constant torque rolling resistance is particularly exposed: at *I* = 10^−3^ and μ_r_ = 0.1, Ω_r_ ≤ 0.7 would require *G*/σ_n_ of order 10^6^–10^7^ with a Rayleigh-based step, i.e., close to or beyond real glass at 10 kPa. Since only the time step was varied here, the threshold Ω_r_ ≈ 0.7 and the dependence on μ_r_, σ_n_, *d* and *I* are estimates that remain to be tested in other configurations. Rolling resistance models with a rotational stiffness and damping [21] have their own stability limit and were not examined.

### 4.2. What Softening Does Change

Once the time step is converged, the friction of our bed hardly depends on the stiffness over two and a half decades, whereas the contact network depends on it strongly. This pattern agrees with two-dimensional simulations across the soft–rigid transition, in which the friction law changes only marginally with stiffness while the coordination number changes strongly [19]. Simulations of weakly frictional grains in slow split-bottom flows found a lower effective friction in softer beds [17]; our data do not exclude such an effect at lower stiffness or with other contact laws, but in the range tested it is below 2% when the time step is converged.

The network statistics respond to the compliance itself. In a soft bed, each contact carries a larger overlap, so near-contacts that carry almost no force are closed, rattlers are recruited into the network, and contacts survive longer before they open (Figure 5). Whether a near-contact is closed or open depends on the overlap scale compared with the gaps between neighbors, so each stiffening step can open a further fraction of them and add rattlers even when the force-bearing skeleton is already settled. These changes do not alter the friction: the extra contacts of a soft bed are oriented more isotropically (smaller *a*_c_), but the friction stays the same (Figure 6d), presumably because the tangential forces carried by the additional contacts compensate. A mechanism in which longer-lived contacts in the extensional direction lower the friction of soft beds is therefore not supported by the time step-corrected data. The granular temperature, which rises with stiffness, has both a compliance part and a time step part; stiffer contacts are briefer, so momentum is exchanged in shorter and sharper events, but a large time step also lowers the translational fluctuations.

### 4.3. Practical Guidance and Limitations

For this cell, stress and material, the results suggest a guide by purpose. When constant torque rolling resistance is used, the friction, the dilatancy, the sliding statistics and the wall torque share require a converged time step, which should be checked by halving the step rather than inferred from the Rayleigh fraction; the impulse ratio Ω_r_ of Equation (10) gives a first estimate, and in this study Ω_r_ ≲ 0.7 kept the friction within 1% of its converged value. With such a step, *G*/σ_n_ = 10^4^ reproduced the friction of the stiffest grains, and even 10^3^ came within about 2%. The solid fraction and the vertical diffusion agree with K300 within 3% from 10^4^. Coordination numbers, rattler fractions, contact persistence and force statistics change with the compliance itself and still differ from K300 at 10^5^, so they should be reported together with the stiffness ratio. The overlap rule alone is insufficient for either channel: it is satisfied at 3 × 10^3^, where the network statistics are still far from K300 and the friction, with a Rayleigh-based step, is 14% low. For comparing frictional or geometric parameter sets, Rayleigh-based steps at 10^4^ reproduced the friction changes of the tested end members to within 0.01, because the time step deficit shifted all of them by nearly the same amount, but they underestimated the effect of rolling resistance itself by about 20%.

The thresholds should not be transferred directly to other conditions. For Hertzian contacts, the state of a packing depends on the stiffness through κ [13], which suggests that the same *G*/σ_n_ thresholds could hold at other normal stresses for the compliance channel. At 100 kPa, for example, the BASE ratio would correspond to *G* = 1 GPa. Keeping *G*/σ_n_ fixed while changing σ_n_, however, does not keep the inertial number, the ratio of the bed weight to the applied load or the ratio of wall to particle stiffness fixed. In addition, the impulse ratio Ω_r_ depends on the time step and the shear rate rather than on *G*/σ_n_ alone. This transfer is therefore a hypothesis that we have not tested. The same caution applies to other materials. The elastic moduli of oxide glasses depend on composition; ultrasonic measurements on doped zinc bismuth borate glasses, for example, show the moduli and Poisson’s ratio rising or falling with the dopant [35]. In the model, the composition of a glass enters only through its elastic constants and density, which set *G*/σ_n_ and so place the glass on the stiffness axis studied here. It also enters through the contact coefficients μ_s_, μ_r_ and *e*. Glasses of different composition would therefore have to be represented by their own values of all of these. The present results say nothing about how composition changes the contact coefficients themselves. Finally, all comparisons in this study are internal verifications of the numerical model. They show how strongly its output depends on the particle stiffness and the time step, but they do not validate the model against laboratory ring shear measurements, which would be required before the absolute friction values are used for a real material.

Several further limitations bound these conclusions. The sweep was run at a single normal stress, with monodisperse spheres in one cell geometry and one rolling resistance model, and the thresholds therefore apply to this configuration and should not be treated as universal values. The time step tests restarted three stiffnesses from a common state and ran for 1 s; the softest run was still 2.1% below the plateau at an eighth of its step, so its time step part is a lower bound. Monodisperse beds crystallize slowly at the platens (Appendix A), and the runs without rolling resistance crystallize faster, so their friction falls by about one-fifth between 3–4 s and 7–8 s (Appendix B). Packing replicates were run for representative cases only, and the trend test covers five parameters at two stiffnesses one factor of three apart, with two of them repeated at 10^5^.

## 5. Conclusions

We varied the stiffness of monodisperse glass spheres with a constant torque rolling resistance in a ring shear cell over two and a half decades of *G*/σ_n_ and separated the effect of the contact compliance from that of the time step, which the Rayleigh rule lengthens as the grains soften. With Rayleigh-based steps, the friction rose by 26% from the softest to the stiffest grains, but restarting the runs with smaller steps removed this rise: at reduced steps the friction agreed within 0.1% from 10^4^ to 3 × 10^5^ and within about 2% at 10^3^, and without rolling resistance it depended on neither the stiffness nor the time step. The friction deficit of the softened runs grows with the angular impulse that the rolling torque delivers in one step and is accompanied by spurious spin fluctuations, so it is a numerical effect of the rolling resistance model rather than a property of soft grains. The dilatancy, the sliding statistics and the sidewall torque share follow the same time step channel. The contact network, by contrast, responds to the compliance itself: the coordination number, the rattler fraction and the contact persistence changed strongly with stiffness at converged time steps and were not settled even at 10^5^, while the solid fraction agreed with the stiffest run from 10^4^. Because the time step deficit shifted the base run and the frictional and geometric end members by nearly the same amount, softened models with Rayleigh-based steps preserved the friction changes caused by sliding friction, sidewall friction and grain size to within 0.008, but they underestimated the effect of rolling resistance by about one-fifth, and the restitution effect vanished between 10^4^ and 10^5^. For this cell, stress and material, the time step of a simulation with constant torque rolling resistance should therefore be checked for convergence separately from the particle stiffness, and the stiffness should be chosen according to the contact network statistics that are of interest rather than by the overlap rule alone.

## Figures and Tables

**Table 1:** Material, contact and loading parameters. Values in parentheses are those of the end member runs.

Item	Parameter	Value
Glass beads	Diameter *d*	1 mm (2 mm)
	Density ρ	2500 kg m^−3^
	Shear modulus *G*	0.01–3 GPa (BASE 0.1 GPa)
	Poisson’s ratio ν	0.2
Steel platens	*G*, ν, ρ	83.3 GPa, 0.2, 7800 kg m^−3^
Teflon sidewalls	*G*, ν, ρ	0.55 GPa, 0.4, 2187 kg m^−3^
Glass–glass	Restitution *e*	0.9 (0.1)
	Sliding friction μ_s_	0.4 (0.1)
	Rolling friction μ_r_	0.1 (0)
Glass–steel	*e*, μ_s_, μ_r_	0.9, 0.6, 0.1
Glass–Teflon	*e*, μ_w_, μ_r_	0.9, 0.04 (0.6), 0.04
Loading	Normal stress σ_n_	10 kPa (*F*_z_ = 25.9 N)
	Lower-platen rotation Ω	3.64 rad s^−1^ (*v* = 0.1 m s^−1^)
	Bed mass; grains	0.0351–0.0353 kg; 26,831–26,957
	Bed mass; grains (*d* = 2 mm)	0.0357–0.0358 kg; 3406–3423
	Upper platen: mass; speed cap	0.105 kg; 2 m s^−1^
	Gravity	9.81 m s^−2^
Numerics	Time integration	Explicit Euler, fixed step
	Time step	20% of the Rayleigh time (sweep); 1/2–1/8 of it (time-step tests)
	Output interval	0.01 s

**Table 2:** Stiffness sweep. κ from Equation (4); δ/*d*, measured mean and 99th-percentile overlap in the comparison window; Δ*t*, Rayleigh-based time step; cost relative to BASE for the same number of grains and simulated time; Ω_r_, per-step impulse ratio of Equation (10) at the Rayleigh-based step (2.2–3.0 s); reduced steps of the runs restarted at 2.0 s, as fractions of Δ*t*.

Run	*G* (GPa)	*G*/σ_n_	κ	Mean δ/*d* (%)	99th δ/*d* (%)	Δ*t* (µs)	Cost	Ω_r_	Reduced Steps
K001	0.01	10^3^	184	1.14	3.27	5.46	0.32	7.85	1/2, 1/4, 1/8
K003	0.03	3 × 10^3^	383	0.60	1.81	3.15	0.55	4.84	—
BASE	0.1	10^4^	855	0.29	0.95	1.73	1.00	2.69	1/2, 1/4
K030	0.3	3 × 10^4^	1778	0.15	0.52	1.00	1.73	1.57	—
K100	1	10^5^	3969	0.07	0.27	0.55	3.16	0.84	1/2
K300	3	3 × 10^5^	8255	0.03	0.15	0.32	5.48	0.47	—

**Table 3:** Time step and compliance parts of the stiffness effects (Figure 7). K300: value of the stiffest run over 2.2–3.0 s; Δ*t* part: Rayleigh-based step minus smallest step; *G* part: smallest step minus K300; both in % of the K300 value, at *G*/σ_n_ = 10^3^ (K001: smallest step 1/8) and 10^4^ (BASE: smallest step 1/4). Groups as in Figure 7b.

Group	Observable	K300	Δ*t* Part, 10^3^	*G* Part, 10^3^	Δ*t* Part, 10^4^	*G* Part, 10^4^
Time step	Spin fluctuation (rad s^−1^)	16.7	+158	−4	+28	−1
	Friction, platen torque μ	0.552	−19	−2	−8	0
	Friction, contact stress μ_σ_	0.495	−19	−2	−8	+2
	Sidewall torque share	0.068	+11	−4	+8	−2
	Force share of sliding contacts	0.211	−48	+15	−20	+6
	Dilatancy Δ*H*/*d*	0.59	−38	+10	−9	+8
Compliance	Strong contact fraction	0.294	+9	+23	+8	+15
	Contact anisotropy *a*_c_	0.146	−10	−24	−5	−10
	Vertical diffusion *D*_z_	0.0441	+1	−12	−3	0
	Coordination number *Z*_m_	3.63	+7	+26	+3	+10
	Coordination number *Z*	2.70	+11	+51	+6	+22
	Rattler fraction	0.224	−8	−75	−9	−39
	Contact persistence	0.647	−1	+29	+1	+14
Mixed	Force anisotropy *a*_n_	0.114	−14	+8	−7	+8
	Granular temperature *T*_g_	1.03	−21	−23	−15	−9
	Solid fraction φ	0.551	+2	+3	+1	0
	Radial diffusion *D*_r_	0.0321	+16	−25	−8	−6
	Force tail exponent β	0.81	+17	+22	+7	+12

**Figure A1:**
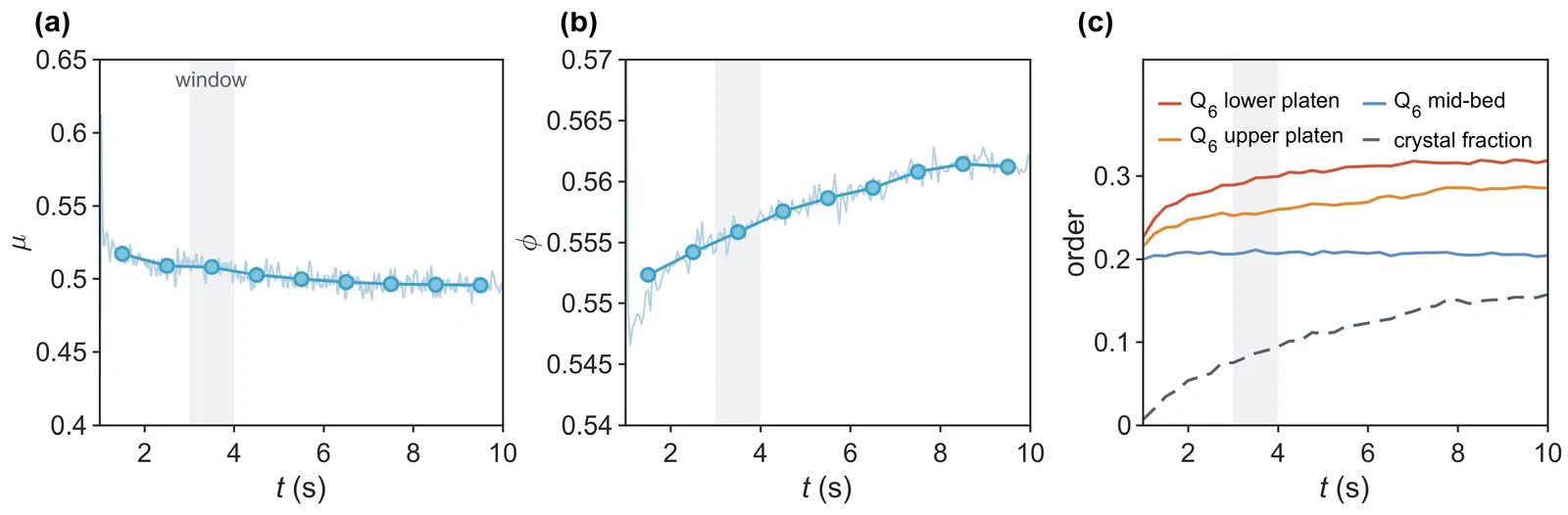
Long-run check of BASE continued to 10 s. (**a**) Platen friction: thin line—0.05 s running mean; symbols—1 s means; shaded—the comparison window used in the main text. (**b**) Solid fraction. (**c**) Locally averaged bond order $\bar{Q}$_6_ within three diameters of the lower and upper platens and in the middle of the bed, and the crystalline fraction of the whole bed ($\bar{Q}$_6_ > 0.4).

## Data Availability

The simulation outputs and analysis scripts that support the findings of this study are available from the corresponding author upon reasonable request.

## Appendix A. Long-Run Check of the Comparison Window

To test how far the 3–4 s window is from a long-time state, BASE was continued to *t* = 10 s with its Rayleigh-based step, i.e., to a nominal shear strain of about 90 (Figure A1). The friction falls slowly, from 0.508 in the 3–4 s window to 0.496 in the 9–10 s window, and is constant from about 8 s onwards (Figure A1a). Over the same period the solid fraction rises from 0.556 to 0.561 (Figure A1b). Both changes come from slow crystallization at the platens: the locally averaged bond order $\bar{Q}$_6_ rises in the layers within three diameters of the upper and lower platens, and the crystalline fraction of the whole bed increases from 8% to 15%, whereas $\bar{Q}$_6_ in the middle of the bed stays at 0.21 (Figure A1c). The 3–4 s window thus overestimates the long-time friction by about 2.5%. Because every run of this study uses the same window and wall crystallization differs only moderately between stiffnesses, this offset is smaller than the effects discussed in the main text; for the friction changes between parameter sets, the window sensitivity is quantified in Section 2.4.

## Appendix B. Numerical Robustness Checks

Four checks were made on the numerical results (Table A1).

*Block length.* For every run, the block standard error of the platen friction was computed with 2, 4, 5, 10 and 20 blocks of the comparison window, together with the integrated autocorrelation time of the detrended series. The autocorrelation time is 0.005–0.009 s in all runs, so even 20 blocks of 0.05 s contain about six autocorrelation times each. For runs without drift, the standard error changes by less than a factor of two between five and 20 blocks; for runs with drift, it decreases as the blocks shorten, because shorter blocks resolve the drift as a trend rather than as a fluctuation, and it falls to 0.0003–0.0007 once the linear trend is removed.

*Packing-to-packing variability.* BASE and the rolling friction end member at *G*/σ_n_ = 10^4^ were each run with three independent packings, and K030 with two. The platen friction of BASE was 0.508, 0.507 and 0.509 (standard deviation 0.0007), that of K030 0.530 and 0.527 (0.0023), and that of the end member 0.320, 0.316 and 0.300 (0.0105). The solid fraction and the coordination number varied by at most 0.002 and 0.02 between packings. The larger spread without rolling resistance comes from crystallization, which proceeds at different rates in different packings. The mean rolling friction effect at 10^4^, −0.196 ± 0.006, agrees with the single-packing value of −0.188 used in Section 3.7. The second K030 packing lies 3.6% below K300, against 3.0% for the first.

*Time step.* The time step tests are described in Section 2.5 and their results in Section 3.2. In addition, K001 and K100 at half their steps were continued to 4 s; over 3–4 s their friction was 11.3% and 1.1% above that of the original runs, the same changes as over 2.2–3.0 s. Continuing K001 from 4 s with its original step produced no change at the restart, so the jumps of Figure 3b are not caused by restarting itself.

*Run length.* K001 was continued to 8 s, and the second packings of K030 and of the rolling friction end member were run to 8 s. Between the 3–4 s and 7–8 s windows the friction fell by 3.3% in K001, 1.7% in K030 and 2.3% in BASE (continued to 10 s), so the deviations of the soft runs from K300 change by only 1.6–2.7 percentage points. Without rolling resistance the friction fell by 22%, from 0.316 to 0.246, as the crystalline fraction grew and the solid fraction rose from 0.596 to 0.611, so the rolling friction effect relative to BASE grew from −0.19 to −0.25. Results for the runs without rolling resistance therefore describe a slowly crystallizing state at a given strain rather than a steady one.

**Table A1:** Numerical robustness checks of the platen friction μ (comparison window 3–4 s unless stated). SD: standard deviation between packings; SE: block standard error.

Check	Runs	Reference	Result
Packing replicates	BASE, 3 packings	—	μ = 0.508, 0.507, 0.509; SD 0.0007
	μ_r_ = 0 at 10^4^, 3 packings	—	μ = 0.320, 0.316, 0.300; SD 0.0105
	K030, 2 packings	—	μ = 0.530, 0.527; SD 0.0023
Time step (2.2–3.0 s)	K001 at 1/2, 1/4, 1/8 of Δ*t*	K001	+11.4%, +19.4%, +23.7%
	BASE at 1/2, 1/4 of Δ*t*	BASE	+5.8%, +8.6%
	K100 at 1/2 of Δ*t*	K100	+1.1%
	μ_r_ = 0 at 10^4^, 1/2 of Δ*t*	μ_r_ = 0 run	−0.5%
	K001 restarted at 4 s, same Δ*t*	K001	no change at the restart
Run length (7–8 s)	K001; K030 (2nd packing); BASE	3–4 s window	−3.3%; −1.7%; −2.3%
	μ_r_ = 0 (2nd packing)	3–4 s window	−22% (crystallization)
Block length	all runs	5 blocks	SE changes by < 2× for 5–20 blocks
